# 1.P. Skills building seminar: Taking the elevator pitch to the next level: how to convince a policymaker in less than 2 minutes

**DOI:** 10.1093/eurpub/ckac129.058

**Published:** 2022-10-25

**Authors:** Organised by: EUPHA, Chair persons: Maaike Droogers (EUPHA), Monica Brînzac (EUPHAnxt)

## Abstract

Since a number of years the European Public Health Association (EUPHA), the European Public Health Conferences and other organisations have been working hard to translate the evidence in a such a format that policymakers take notice. For example, the WHO Regional Office for Europe works on ‘telling the public health narrative’ and provides factsheets and infographics, in order to effectively communicate public health messages to policymakers. At the European Public Health Conference so-called pitch presentations were introduced (at Glasgow 2014), where researchers are asked to present their work in 5 minutes with maximum 5 slides (no animations), as a way to learn to present key messages from research in just a few minutes. EUPHA has organised several skills building workshops on translation of evidence in the past years, including the 2019 session ‘making the elevator pitch work’, then in 2020 ‘making the elevator pitch more effective’, and last year ‘making the elevator pitch perfect’. Building forward on those three successful and well-attended workshops, the current workshop will follow up on this series and dive deeper into communicating the evidence through the elevator pitch. Lessons learned at the previous elevator pitch workshops:

– Have a clear ask (keep it simple).

– Appeal to the policymaker's own interests and priorities.

– Spell out how action will be beneficial for the policymaker.

– Be aware of upcoming elections.

– Built a relationship with the assistants of politicians.

– Considering the ‘policy window’.

– Make the comparison with the policy plan.

– Propose an action the politician should undertake.

– Identifying the relevant stakeholders and groups affected by the problem.

The importance of effectively communicating the evidence to policymakers is highlighted by infodemics, e.g. called out by the WHO and UN in the context of the COVID-19 pandemic with the spreading of mis- and disinformation about the pandemic. Considering the physical distancing measures that were in place the past years, which made teleworking (working from home) more common, the workshop will also cover virtual tactics. Communicating the science in an increasingly virtual world has made it a whole different kind of sports. In this skills-building workshop, we will select a number of abstracts that have been accepted by the International Scientific Committee as posters and we will invite the presenting authors to this dare: Present your work and key messages in less than 2 minutes. In order to see whether the policymaker/politician is convinced, we are organising a small panel of policymakers and politicians and ask them to give their feedback. Are they interested? Do they remember the key message? And if all goes well, do you get an invitation to come back and present more of your work?

**Key messages:**

• Being able to present your key messages anywhere, anytime is needed (including virtual tactics).

• Telling the public health narrative and telling a story are important skills for public health professionals to have.

**Speakers/Panellists:**

**Marleen Bekker**

EUPHA (PHPP)

